# c-Src regulates cargo transit via the Golgi in pancreatic acinar cells

**DOI:** 10.1038/s41598-018-30370-4

**Published:** 2018-08-09

**Authors:** Sergiy Kostenko, Chan C. Heu, Jordan R. Yaron, Garima Singh, Cristiane de Oliveira, William J. Muller, Vijay P. Singh

**Affiliations:** 1 Department of Medicine, Mayo Clinic, Scottsdale, Arizona USA; 2 Goodman Cancer Research Center and Department of Biology, McGill University, Montreal, QC H3A 1A3 Canada

## Abstract

The exocrine pancreatic acinar cell is unique for its rapid protein synthesis and packaging in zymogen granules (ZGs). However, while crucial to the pathogenesis of pancreatitis, the signaling involved in the transit of proteins via the Golgi is poorly understood in these cells. Noting the evidence of c-Src in regulating transit of cargo via the Golgi in other systems, we explored this in acinar cells. Stimulation of ZG formation with dexamethasone activated Src and increased the Golgi area in acinar cells. c-Src localized to the microsomes of acinar cells on immunofluorescence and subcellular fractionation. While other Src family members had no effect on the Golgi markers P115 and GM130, active c-Src increased the Golgi area these stained, extending them into the ER. Src inhibition reduced amylase staining outside the Golgi and increased it in a stack like Golgi morphology. *In vivo* pharmacologic inhibition or acinar specific genetic deletion of c-Src reduced ZG number and staining of amylase in ZGs along with increasing amylase retention in the microsomal fraction. Morphologically this was associated with smaller Golgi stacks, and dilation of the endoplasmic reticulum. Therefore the role c-Src regulated Golgi function, ZG formation and microsomal zymogen transit in acinar cells needs to be explored in pancreatitis.

## Introduction

While pancreatic acinar cells have one of the most rapid protein synthetic and packaging machineries^1^, the mechanisms regulating zymogen granule (ZG) formation- the organelles in which these proteins are stored for regulated secretion are not well understood. At the junction of the protein synthetic machinery of the endoplasmic reticulum (ER) and ZG formation lies the Golgi, where proteins targeted for secretion are sorted and packaged in vesicles- the immature secretory granules- which mature to ZGs^2^. Several studies have shown that the Golgi of pancreatic acinar cells is a network of anastomotic, branching elongated ribbon like structures^3,4^ referred to as cisternae. The transport of cargo and resident proteins to and from the ER to the Golgi is complex and while not studied in detail in pancreatic acinar cells, has been shown to involve maturation of Golgi cisternae, along with retrograde vesicular flow of Golgi resident proteins into the ER^5^. We have previously shown that Arf-1 protein is involved in antegrade transport and maturation of the lysosomal enzyme cathepsin B through the Golgi, and pharmacologic Arf-1 inhibition to result in accumulation of its precursor pro-cathepsin B, reduced autophagic maturation, trypsinogen activation and severity of pancreatitis^6^. Src has been shown to reside on the Golgi^7^ and Src activation in acinar cells results in actin reorganization, trypsinogen activation along with vesciculation of the Golgi, resulting in cell injury^8^. Since Src activation was shown to cause redistribution of Golgi resident proteins including N-acetylgalactosamyl transferases into the ER in non-secretory cell types such as the HeLa cells and W138 fibroblasts via Arf-1^9^, and regulate transit through the Golgi^10^ and trans-Golgi network (TGN) via dynamin-2^11^; we chose to study the role of Src in Golgi dynamics in a rapidly secretory cell type- the pancreatic acinar cell. The cargo we studied is amylase, an abundant exocrine protein that is packed in zymogen granules and secreted in a polarized manner. This was also chosen to avoid the alternate trafficking of lysosomal hydrolases into lysosomes and retrograde transport of ER resident proteins with a KDEL sequence^10^.

While several Src family kinases have been identified in acinar cells including c-Src^12^, Yes^13^, Lyn^14^ and Fyn^15^, which have been known to regulate the actin cytoskeleton^13,15^, adherens junction^16^, endocytosis^12^, cytosolic calcium signaling^17^ in addition to secretion, and trypsinogen activation^8^; the tools used to study these have heavily relied on pharmacologic inhibition which is not specific for individual Src family members. We therefore chose to identify the family member(s) involved in amylase trafficking through the Golgi using subcellular fractionation, over expression of Src family members, along with genetic knockdown in the adult mouse. Our data show that c-Src is present on the Golgi and ER of acinar cells and its activity regulates transit of amylase along the secretory pathway.

## Results

### c-Src is present on the Golgi and ER of acinar cells

**Figure 1:**
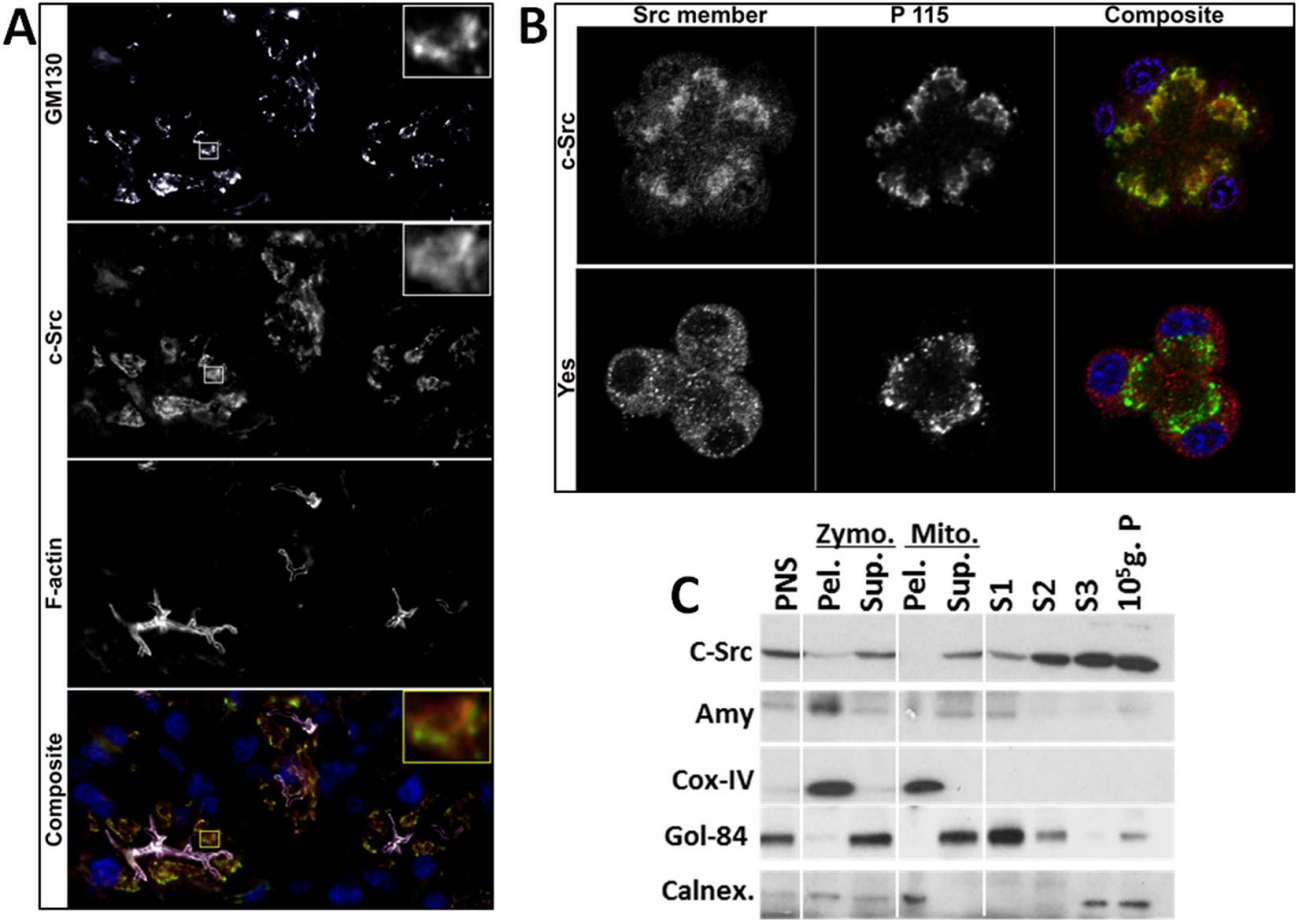
Organellar Localization of c-Src by immunofluorescence and subcellular fractionation. (**A**) Top to bottom**:** Immunofluorescence in cryosections of C57BL/6 mouse pancreas for GM130, c-Src and F-actin along with a composite image at the bottom. Inset at the right upper corner are magnified views of the area outlined in squares. (**B**) Immunofluorescence in mouse pancreatic acini with another Golgi marker P115 stained for c-Src (upper panel) and Yes (lower panel). Note the co-localization of the Golgi markers with c-Src, which also extends beyond the Golgi into the ER. (**C**) Western blot of sub-cellular fractions of mouse pancreas separated into post nuclear supernatant (PNS), the Zymogen (Zymo.) pellet (Pel.) enriched in amylase and supernatant (Sup.), mitochondrial (Mito.) pellet enriched in COX-IV and supernatant, and microsomal fractions containing the Golgi marker Golgin-84 (Gol-84) enriched in S1, S2 and the ER marker calnexin enriched in the S3, 10^5^ g. pellet fractions. Note c-Src is enriched in the microsomal fractions.

We first determined the subcellular location of c-Src. Staining of endogenous c-Src in mouse pancreatic tissue showed this to predominantly co-localize with the cis-Golgi marker GM 130 in exocrine acinar cells (Fig. 1A), though it extended both apically and basally around it. This was verified with another Golgi marker P115 in isolated acinar cells (Fig. 1B), and while the two did co-localize, c-Src also showed a diffuse pan cytoplasmic appearance consistent with the endoplasmic reticulum (ER). Interestingly this localization was not noted for the Src family member Yes, which has been previously shown to be present in acinar cells. On sub cellular fractionation c-Src was enriched in the microsomal fractions in the 10000 g supernatant which contained both Golgi and ER (Fig. 1C). Subcellular fractionation of pancreatic tissue showed c-Src to enrich in the Golgi and ER fractions (Fig. 1C) which were respectively marked by the resident proteins Golgin-84 (Gol-84) and Calnexin (Calnex.) which lacks a KDEL sequence.

### c-Src activation results in the Golgi expansion

**Figure 2:**
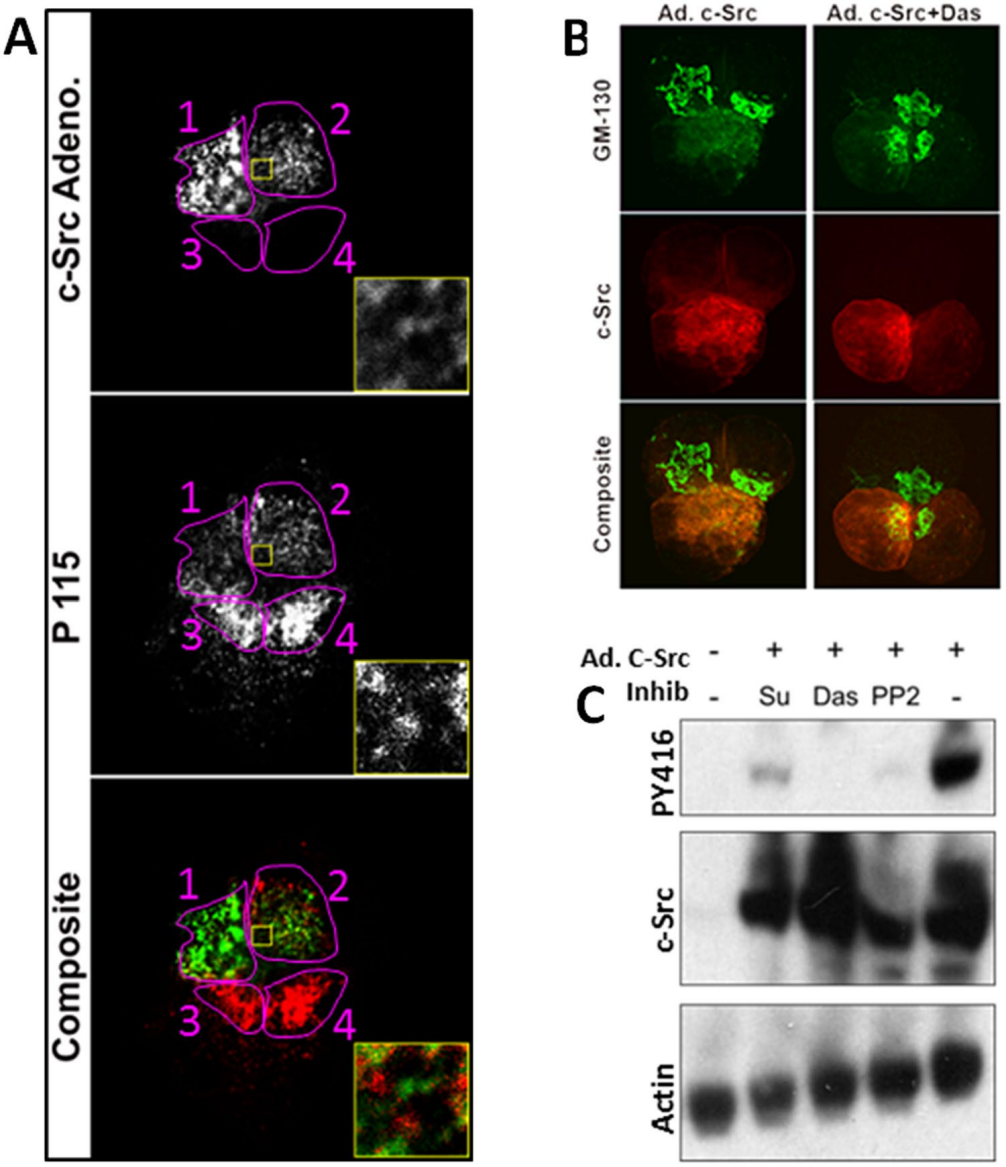
Effect of c-Src overexpression on Golgi morphology. (**A**) Primary mouse pancreatic acinar cells expressing c-Src adenovirus (Cells 1, 2) are shown in the top panel. The effect of this on the Golgi marker p115 is shown in the middle panel and composites in the lower panel. Inset magnified areas showing the hollow unstained round areas containing zymogen granules and both c-Src and P115 are extending into the basal ER. (**B**) AR42J cells infected with c-Src adenovirus alone (left panel) or with the Src inhibitor Dasatinib (Das; 10 μM) in the right panel. In Green is the golgi marker GM-130 (upper panel), red is staining for c-Src (middle panel), with the composite image shown in the lower panel. Note Src inhibition with Dasatinib prevents the extension of GM130 staining in the c-Src expressing cell. (**C**) Western blot of AR42J cell lysates overexpressing adenoviral c-Src blotted for active Src (PY416) upper panel, c-Src (middle panel) and loading control actin (lower panel) showing the effects of pharmacologic inhibition of Src with SU6656 (Su), Dasatinib (Das) and PP2 on Src activation.

To study the effect of c-Src activation *in vitro*, we first adenovirally over expressed c-Src in primary pancreatic acinar cells. Low levels of Src expression (Fig. 2A, cell2) resulted in an increase in P115 positive area compared to the untransfected cells (cells 3,4). This increase appeared in the cytoplasm/ER around unstained round areas resembling zymogen granules (inset yellow squares). This was also noted in AR42Jcells (Fig. 2B) where the Golgi marker GM130 behaved similarly. Interestingly, high level of Src expression (also seen in cell 1, Fig. 1A) diminished the Golgi marker staining intensity compared to un-transfected cells in which the Golgi remained compact (Fig. 2C left column). These effects of c-Src were dependent on its activation since pharmacologic Src inhibition with different inhibitors (Fig. 2C, SU6655; 10 μM, Dasatinib 10 μM, and PP2 10 μM) prevented its activation and the changes in Golgi morphology (Fig. 2B).

**Figure 3:**
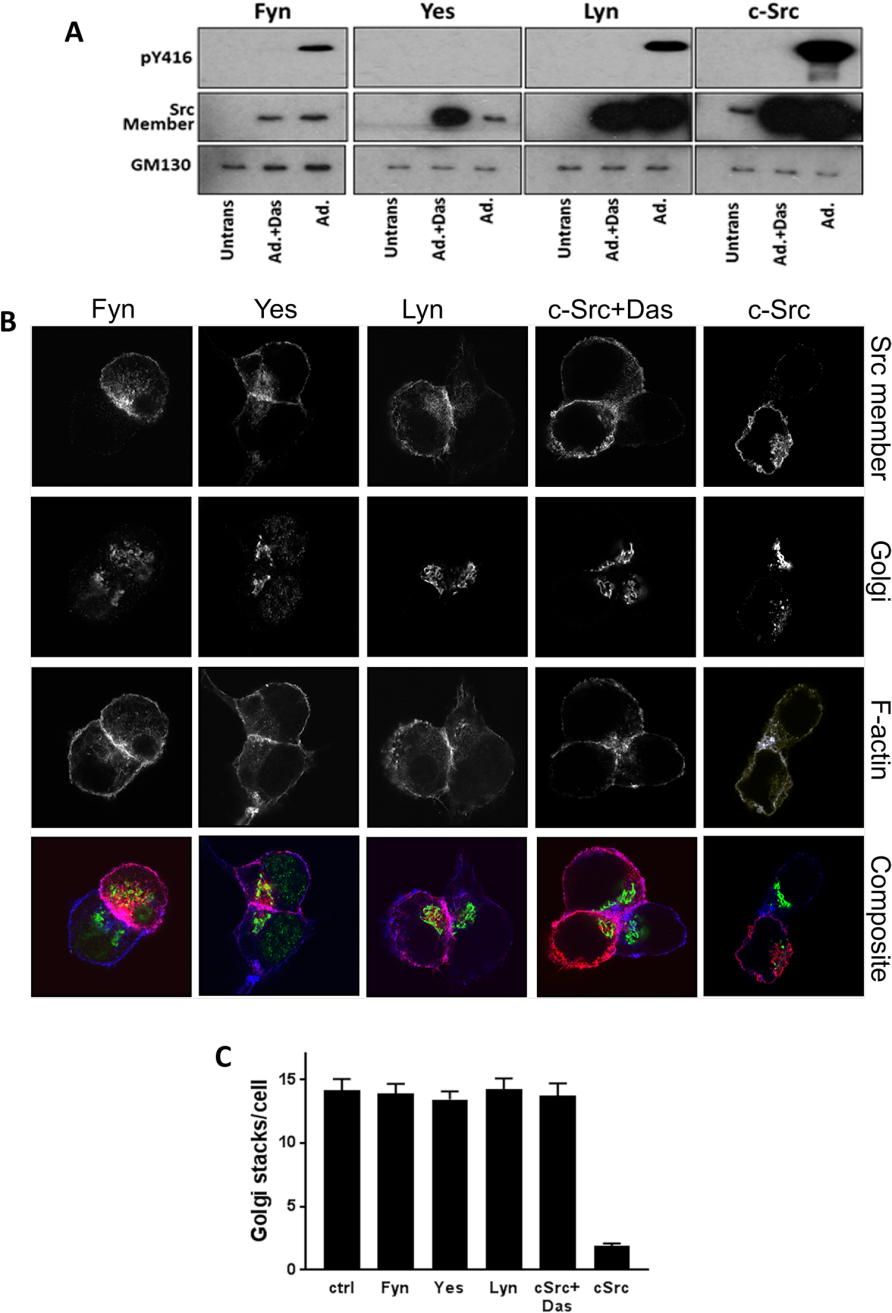
Effect of overexpression of different Src members on Golgi morphology. (**A**) Western blot of AR42J cell lysates overexpressing Fyn, Yes, Lyn and c-Src adenovirally with active Src (PY416) upper panel, Src family member (middle panel) and GM-130 as lower panel. Note that Yes is not activated on overexpression, and Dasatinib (Das) prevents Src activation. (**B**) Immunofluorescent images of adenovirally expressed Src member (upper panel), Golgi (second panel), F-actin (third panel) and composite image (Lower panel). Note, c-Src expressing cells has complete fragmentation of the Golgi, which was prevented by Dasatinib. (**C**) Bar graphs displaying the number of individual Golgi stacks in AR42J cells overexpressing Src members vs control.

We next went on to study the Src family members that affected Golgi morphology. While Fyn, Yes, Lyn and c-Src could be overexpressed adenovirally (Fig. 3A), Yes was not spontaneously activated as noted by western blotting against the active form (pY416). Interestingly while all members showed a tropism for the Golgi area (Fig. 3B) and the cell membrane, only c-Src caused the morphological change in the Golgi (Fig. 3B,C), which were prevented by Dasatinib.

### Pharmacologic inhibition of Src reduces granule formation in AR42J cells

**Figure 4:**
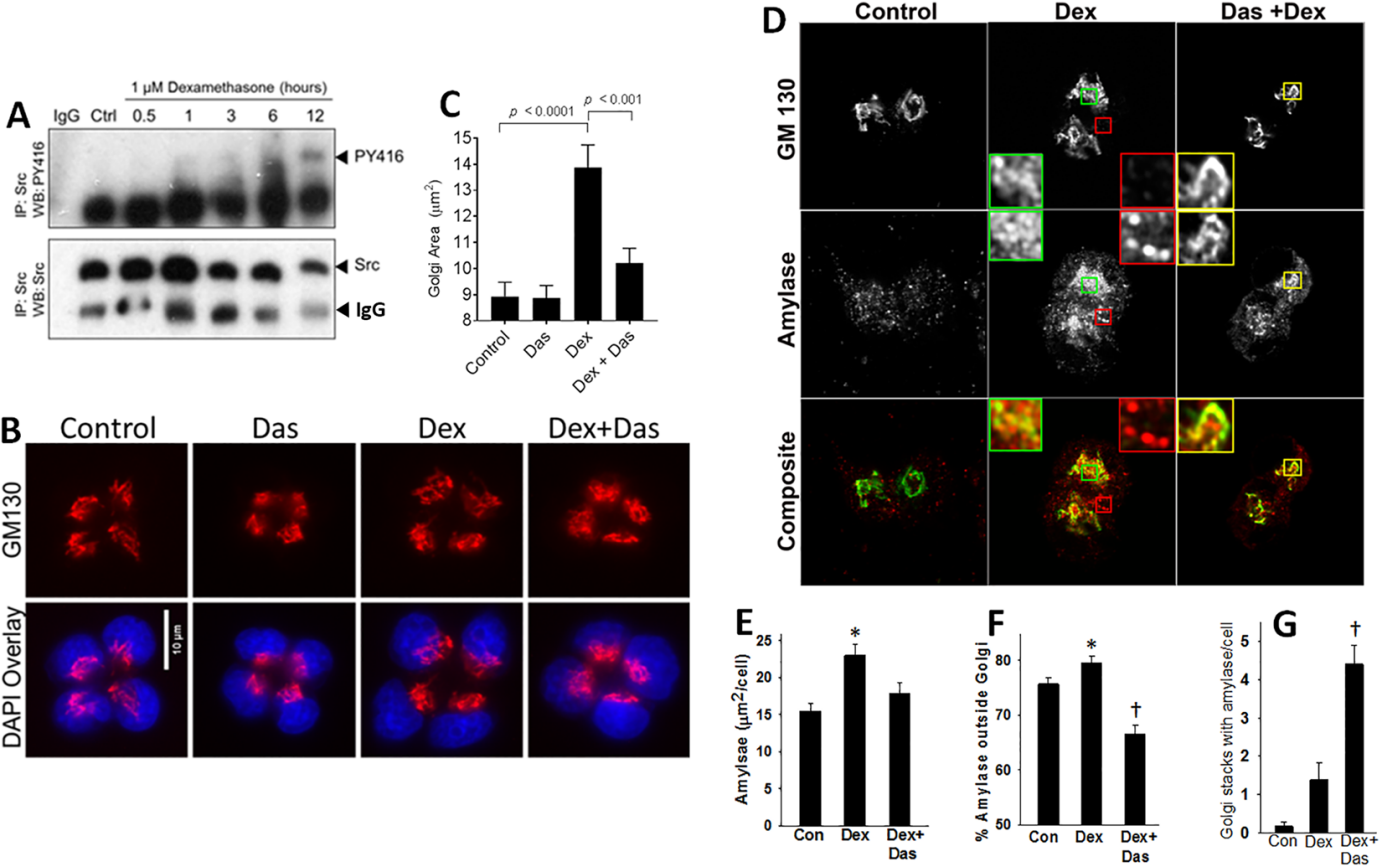
Effect of pharmacologic Src inhibition on dexamethasone (Dex) induced changes in AR42J cells. (**A**) Western blot of AR42J cell lysates collected after differ times of Dex (1 μM) stimulation immunoprecipitated for total Src and blotted for active Src (PY416; upper panel) and total Src (middle panel). (**B**) Immunofluorescent images of AR42J cells after one day stimulation with Dex stimulation stained for GM130(upper panel) with DAPI overlay (Lower panel). (**C**) Bar graph of Golgi area of AR42J cells after different treatments. (**D**) Immunofluorescent images of AR42J cells after 1 day of Dex alone or with Dasatinib (Das) stained for GM 130 (upper panel), amylase (middle panel) and composite images lower panel. Bar graphs quantifying the total stained amylase area (**E**) % amylase outside the Golgi area (**F**) and number of stacks positive for amylase in each cell (**G**). *Indicates a significant increase vs. control and † a significant (p < 0.05) change from Dex induced by Dasatinib.

We then went on to study the role of Src in Golgi dynamics, along with trafficking of the exocrine enzyme amylase. We used dexamethasone (Dex; 1 μM) as previously^18^, which is known to increase amylase amounts. Dex caused Src activation (Fig. 4A). This was associated with an increase in Golgi area from 8.9 ± 3.5 µm^2^ to 13.9 ± 5.9 µm^2^ (p < 0.0001) which was reduced to 10.21 ± 3.6 µm^2^ (p < 0.001) by Dasatinib (Fig. 4B,C).

To study the role of Src in the trafficking of amylase, AR42J cells were starved overnight. Next morning normal medium was replaced with alone Dexamethasone (Dex group) or after pretreatment with 10 micromolar Dasatinib (Dex + Das) and followed for another 24 hours. Dex resulted in a significant increase (*) in total amylase staining (Fig. 4D,E) most of which was round and granular (inset boxes in middle panel of Fig. 4D) along with being outside the Golgi (Fig. 4F). Dasatinib reduced the granular staining outside the Golgi († in Fig. 4F) along with increasing the amylase retained in the Golgi stacks († in Fig. 4G, and right most column in Fig. 4D, highlighted the yellow inset squares). It is to be noted that despite the close proximity of Golgi and granules in the Dex group, majority of the granules did not co-localize with the Golgi (Green inset squares) or were outside the Golgi (red inset squares).

### Genetic deletion or pharmacologic inhibition of c-Src *in vivo* reduces zymogen granule formation

**Figure 5:**
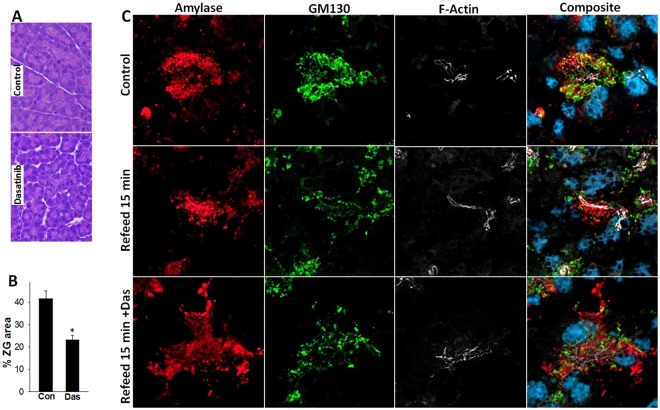
Effect of Pharmacologic inhibition of Src *in vivo* on pancreatic acinar zymogen granule area and amylase staining. (**A**) Bright field imaging of histological sections of C57BL/6 mouse stained with hematoxylin and eosin with no treatment (control) and treatment with Dasatinib. Note the lesser amount of pink (zymogen granule area in with Dasatinib treatment. (**B**) Bar graph showing the % acinar cell area occupied by zymogen granules in the control and Dasatinib treated mouse pancreas. (**C**) Immuno fluorescence of amylase (red), GM130 (green), F-actin (white) and composite in normal fed mice (control; upper panel), mice fasted for 48 hours followed by 15 minutes of refeeding (middle panel) and mice treated with Dasatinib 3 hours prior to refeeding. Note, the diffuse cytoplasmic staining of amylase in the Dasatinib treated mice.

We first studied the pharmacologic effects of Src inhibition as a rapid relevant *in vivo* approach to study the effect of Src on ZG formation. 2 days of Dasatinib treatment reduced the zymogen granule area from 41.6 ± 12% to 23.1 ± 6.3% (p < 0.01) in C57bl/6 mice (Fig. 5A,B). Normal C57bl/6 mice showed amylase staining to be intense in zymogen granules (Fig. 5C upper row, 5D). While refeeding after 48 hour fasting resulted in a return of punctate amylase staining in zymogen granules within 15 minutes, those treated with Dasatinib prior to refeeding had large portion of amylase stain in a diffuse cytoplasmic pattern.

**Figure 6:**
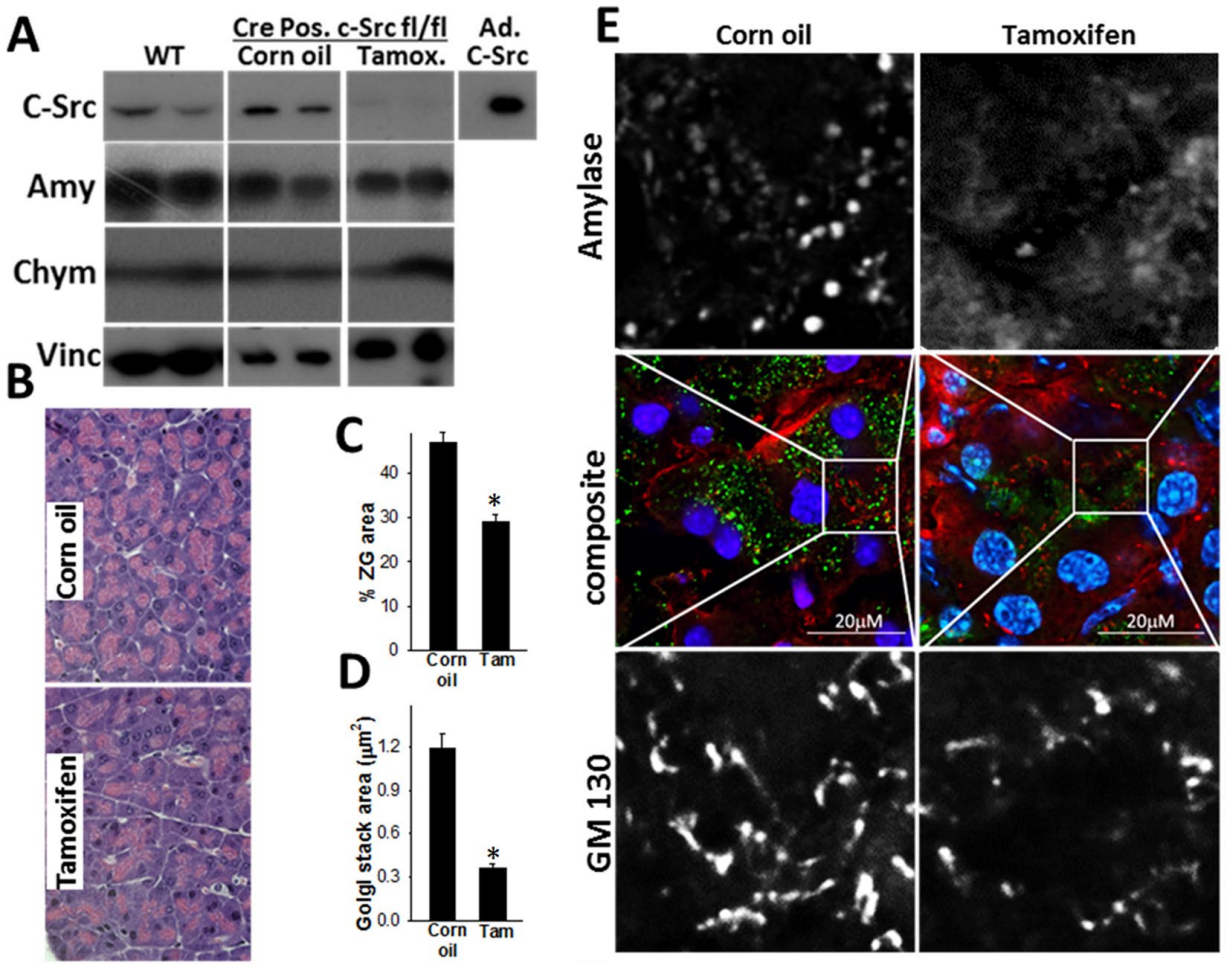
Effect of genetic deletion of c-Src in the exocrine pancreas on zymogen granule, Golgi area and amylase staining. (**A**) Western blots of mouse pancreatic homogenates from C57BL/6 (wild type; WT) mice, Cre positive, c-Src^L/L^ mice given corn oil or Tamoxifen blotted for c-Src (upper panel, with c-Src adenovirus as a positive control), Amylase (Amy), Chymotrypsin (Chy) and vinculin (Vinc) as a loading control. (**B**) Bright field imaging of histological sections of Cre positive, c-Src^L/L^ mice stained with hematoxylin and eosin given corn oil or Tamoxifen. Bar graphs depicting the zymogen granule area as percentage of total acinar cell area (**C**) and area of individual Golgi stacks (**D**) in corn oil or tamoxifen (Tam) treated mouse acinar cells in the pancreas. (**E**) Immunofluorescence images of mouse pancreas stained for amylase (green) and GM130 (red) in corn oil and Tamoxifen treated mice. The greyscale images in the top and bottom row show amylase and GM130 staining of the inset boxes respectively. Note the diffuse amylase staining and smaller Golgi stacks in the Tamoxifen treated mice respectively.

To specifically study the role of c-Src in zymogen granule formation, we genetically deleted it in adult (12–16 week mice) by treating c-Src^L/L^/Cre positive mice with tamoxifen (Fig. 6A). c-Src deletion did not affect the amount of amylase or chymotrypsin protein compared to the vehicle of tamoxifen (corn oil), but resulted in a decrease in the pink staining zymogen granule area in exocrine acinar cells from 46.8 ± 7.1% to 29 ± 4.6% (*p* < 0.01; Fig. 6B,C) similar to the Dasatinib treated mice. On immunofluorescence staining, this was seen as amylase having a diffuse cytosolic staining vs. the normal dense granule staining seen in mice without c-Src knock down (Fig. 6E) or wild type mice (Fig. 5C, upper row). c-Src knockdown also resulted in smaller Golgi stacks (Fig. 6D) with morphologically less branching (Fig. 6E lower panel, right side).

**Figure 7:**
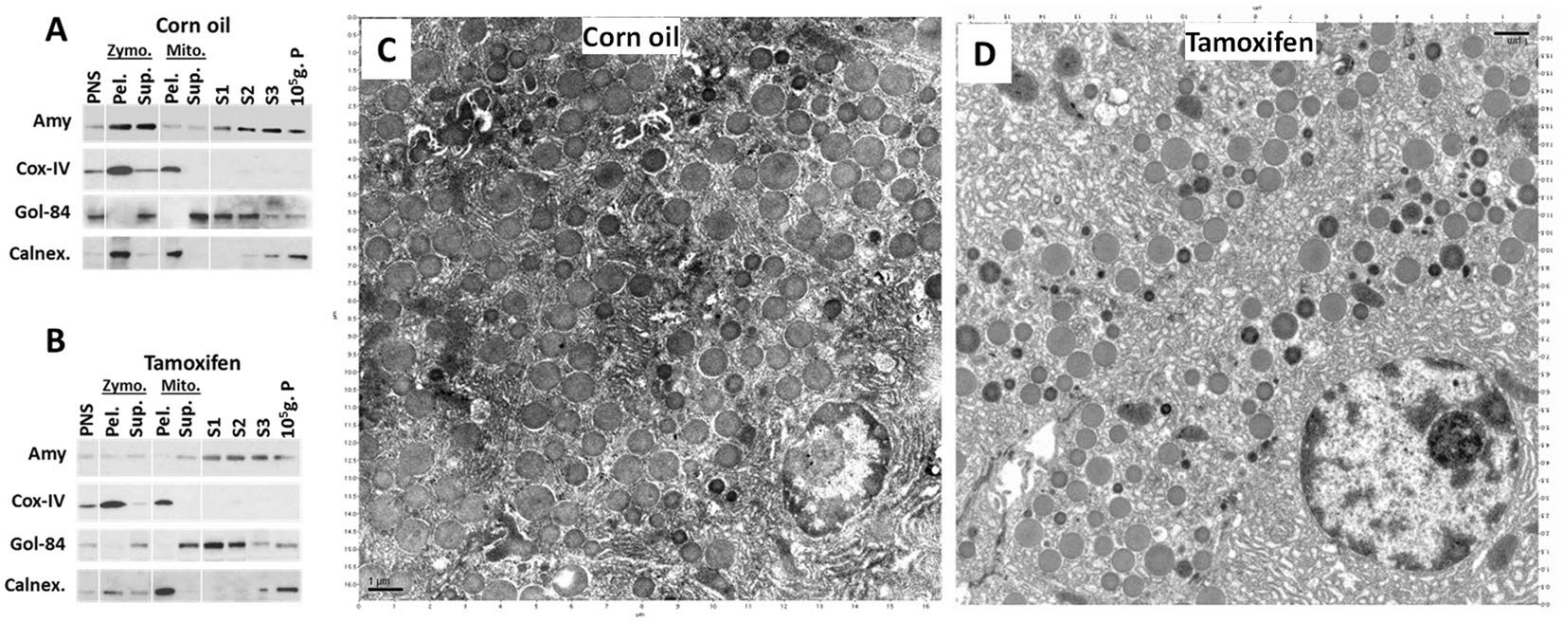
Effect of c-Src knock down on Subcellular localization of amylase and ultrastructure of the pancreatic acinar cell. Subcellular fractionation of pancreas of corn oil (**A**) and tamoxifen (**B**) treated mice, blotted for amylase (Amy), along with the Golgi marker Golgin-84 (Gol-84) and ER marker calnexin (Calnex.). Note that tamoxifen causes loss of amylase in the zymogen fraction along with an increase in the microsomal (S1–10^5^ g.P) fractions. Electron micrographs of acinar cells in the pancreas of corn oil (**C**) or Tamoxifen (**D**) treated mice. Note tamoxifen causes an increase in smaller size of zymogen granules, along with an increase in dilated ER.

On subcellular fractionation, c-Src knockdown resulted in loss of enrichment of amylase in the zymogen granule fraction normally seen without c-Src knockdown, such as in the corn oil treated group (Zymo.; Fig. 7B, vs. 7A). The knock down caused a concurrent increase of amylase in the microsomal fraction (S1 to 10^5^ gP) where the ER and Golgi markers Golgin-84 and Calnexin are present. This data correlates well with the loss of amylase staining in zymogen granules induced by tamoxifen (Fig. 6E). On electron microscopy (Fig. 7C,D), the c-Src knockdown mice has a decrease in zymogen granule number, and an increase in the proportion of small size granules, along with the ER dilation. These findings support the role of c-Src in trafficking of amylase from the Golgi and ER into zymogen granules and in the formation of zymogen granules in acinar cells.

## Discussion

In this study we investigated the role of c-Src on Golgi function and the trafficking of amylase, an exocrine enzyme secreted by pancreatic acinar cells. To do so we first identified the c-Src as a family member that is present on the microsomes of acinar cells. While other Src family members, i.e. Yes, Lyn and Fyn could be overexpressed, these did not perturb Golgi morphology. C-Src overexpression however resulted in morphological expansion of the Golgi markers GM-130 and P115 into the ER. This phenomenon was dependent on c-Src activation and was prevented by inhibiting it pharmacologically. We go on to show that stimulation of ZG formation in AR42J cells is associated with Src activation and expansion of the Golgi area also, and that pharmacologic inhibition of Src results in a reduction of amylase outside the Golgi and an increase in its retention in the stack like morphology of the Golgi. Similarly *in vivo* both pharmacologic inhibition of Src and its selective ablation in acinar cells of adult mice caused retention of amylase in the microsomes, dilation of the ER and a reduction in ZG number. These findings along with studies showing that patients on Dasatinib have a reduced risk of pancreatitis^19^, add a new dimension to the role of Src family members in the acinar cell, and suggest Src as a plausible regulator in pancreatitis.

Previous studies have shown Src family members to regulate upstream signaling in acinar cells including store operated calcium entry^17^, protein kinase c- delta activation via Lyn^20^, physiologic phenomena including endocytosis^12^, exocytosis^13^ and pathological phenomena including blebbing^15^, trypsinogen activation^8^ and chemokine upregulation^21^. In most of these studies however the Src family member responsible for the biologic phenomenon has remained unclear since the approach has traditionally been based on pharmacologic inhibition, which affects all Src family members. The current study provides the first specific role of a Src family member, i.e. c-Src in pancreatic acinar protein trafficking.

Src Kinases have been shown to reside on the Golgi^7,22^, and Src has been shown to be activated via transit of secreted cargo from the ER to the Golgi^11^. Src activation has also been shown to result from ER lumen proteins with the KDEL sequence reaching the cis-Golgi and binding the KDEL receptor, initiating a COP-1 sequence^23^ or intra-Golgi trafficking^10^. While some studies show Src activation to result in fragmentation of the Golgi^11^, others show a redistribution of N-galactosylaminotranferases from the Golgi to the ER^9^ regulating O-glycosylation, without affecting cis-Golgi markers such as GM130. Here we note Src to affect both Golgi dynamics and cargo transit- i.e. c-Src activity induced fragmentation of the Golgi and Src inhibition caused accumulation of amylase (a normally secreted protein without a KDEL sequence) in the ER, Golgi- with resultant ER dilation. The mechanism of this remains unknown. Previous studies inhibiting O-glycosylation showed a reduction of ZG formation in acinar cells^24^ due to impaired condensation of secreted proteins in the TGN. Whether this kind of a phenomenon or a Src regulated cytoskeletal or budding event on the Golgi results in the finding we note remains to be studied. Knowing these can impact both the physiologic modulation of acinar function such as during fasting and refeeding, along with helping us understand the therapeutic relevance of targeting Src during pancreatitis.

## Methods

### Animals and animal procedures

C57BL/6 mice were purchased from Jackson Laboratory (Bar Harbor, Maine) and used at 12–16 week age. These were housed with a 12-h light/dark cycle, at temperatures from 21–25 °C, fed standard laboratory chow, and allowed to drink *ad libitum*. Animals were acclimatized for at least 2 days before use. For genetic deletion of c-Src in the exocrine pancreas, mice with LoxP sites located 5′ of intron 4 and intron 7 of c-Src (NCBI NM_009271) with an FVB/NJ background^25^ were backcrossed to a C57BL/6 background over 5 generations using the 150–180 SNP panel selected from publically available databases. This was done at the Jax Genome Scanning Service (Jackson Laboratory Bar Harbor, Maine). Dual floxed (c-Src^L/L^ mice) were identified using the primers (Forward: GGTCTTGTCATGGCTCTGTC, reverse: CATCTCTGCTCACCTGATAG) yielding a single 450 BP band, wild types yielding a 400 BP band as described previously^25^. c-Src^L/L^ mice were then bred to mice expressing the fusion product of Cre recombinase and a mutant human estrogen receptor ligand binding domain (CreERT2) under the elastase1 promoter [Tg(Ela1-Cre/ERT2)1Stof/J, Jackson labs, Stock # 08861], with the mice expressing Cre recombinase yielding a 500 BP band using CTCTGCTAACCA TGTTCATGCCT as forward primer and ACG CTAGAGCCTGTTTTG as reverse primer. Genetic deletion of c-Src was achieved by administration of tamoxifen (150 mg/kg, in 0.1 ml corn oil, intraperitoneal) to c-Src^L/L^ Cre positive mice as 7 injections over 14 days followed by a 1 week recovery period. This was optimized based on 5 injections with 5 day recovery period at the same dose being ineffective in knocking down the c-Src protein. The control group received only corn oil. At the end of the recovery period, the mice were euthanized using carbon dioxide and the pancreas was immediately procured and used for histological, morphological, biochemical assays as described below. Dasatinib (50 mg/kg/day × 2 days) was given intraperitoneally as described previously^26^. *In vivo* studies are representative of 6–8 animals in each group.

### Adenoviral production

The cDNA sequences corresponding to mouse c-Src (NM_009271.3), Fyn (NM_001122893.1), Lyn (NM_001111096.1) and Yes (NM_009535.3) were cloned into pAdlox vector to generate constructs. Adenoviruses of these constructs were generated at the Vector Core Lab, University of Pittsburgh (Pittsburgh, PA). 4 × 10^8^ plaque-forming units /ml of recombinant adenoviruses were infected in mouse primary acinar cells or AR42J cells for overexpression of the Src proteins and studying their amounts or localization after overnight (16–20 hour) culture.

### Cell lines and pancreatic acini

Primary pancreatic acini were harvested as described previously^15^ from mice and cultured in RPMI 1640 with 10% Fetal bovine serum (FBS; GE life sciences, Logan, UT) at 37C in a 5% CO_2_-humidified incubator^27^ alone or with adenoviruses. Viability next morning before use was more than 95% by trypan blue exclusion. AR42J cells (American Type Culture Collection no. CRL-1492) were cultured in F-12K in 10% FBS with incubator settings as in primary acini. These were used for studies on ZG formation since primary acinar cells do not make ZG in culture. Dexamethasone (1 μM) was used to induce protein synthesis and zymogen granule formation as described previously^28^. Results of *in vitro* studies are reported as averages from at least 3 independent experiments.

#### Reagents

Specific antibodies were GM130, p115, Golgin 84, calnexin (BD Transduction Laboratories, Lexington, KY), TGN 38, Amylase (Sigma, St. Louis, MO), c-Src, Yes, Lyn, Fyn (Cell signalling Technologies, Danvers, MA), Cox-IV (ThermoFisher, Waltham MA), Vinculin (Santa Cruz Biotchnology, Dallas, TX). Dasatinib was from LC Labs (Woburn, MA). All other reagents and chemicals were purchased from Sigma.

### Western Blotting

Proteins were extracted from pancreas or cells after homogenization with a Potter-Elvehjem homogenizer in homogenization buffer containing 50 mM Tris at pH 7.2, 150 mM NaCl, 0.5 mM EDTA, 1 mM EGTA, 2 mM dithiothreitol, 1 mM Na_3_VO_4_, 25 mM NaF, 1% NP-40, and Complete (Roche Diagnostics, Indianapolis, IN) protease inhibitor cocktail. Immunoprecipitation of Src and blotting for active Src was done as described previously^15^. For blotting, lysates were boiled in 1x Laemmli sample buffer before Western blot analysis according to standard procedures as previously described^15^.

#### Subcellular fractionation

This was done on whole pancreatic homogenates as described previously^6^ to yield the zymogen and mitochondrial fractions. The mitochondrial fraction supernatant was further fractionated into the Golgi (S1, S2) and ER fractions (S3, Pellet) as described in the protocol by Taylor *et al*.^29^. Proteins were quantified in these and adjusted to a final concentration of 0.1 μg/μl, boiled in Laemmli buffer for western blotting. The full length un-cropped images are presented in the Supplementary Information.

### Immunofluorescence Microscopy

Immunofluorescence microscopy was done on pancreatic tissue cryosections embedded in Tissue-Tek® Optimal Cutting Temperature (OCT) (Sakura Finetek USA, Inc., Torrence, CA), AR42J cells or acini plated on plain glass coverslips. These were fixed with 2% paraformaldehyde and processed as described previously^15,30^. After blocking with 5% normal goat serum, tissue cryosections were incubated with primary antibodies (1:50 for cryosections and 1:200 for cells) for 1 h at room temperature, washed, and incubated with secondary antibodies (Alexa 488- or Alexa 594-conjugated, diluted 1:500) Cy5-conjugated phalloidin (100 nM) (ThermoFisher, Waltham, MA) with or without DRAQ5 (1:5000) for 30 min. After washing and mounting in Fluoromount G (Sigma Aldrich) on SuperFrost slides (ThermoFisher), confocal imaging (1 µm thick) was done using a Zeiss LSM800 confocal microscope (Thornwood, NJ). Images were collected with a Zeiss C-Apochromat 63x/1.2 NA water immersion objective.

### Quantitative morphometry

Images were acquired and saved as. czi files. Golgi area, total amylase area, and amount of amylase out of the Golgi were quantified using automated image cytometry performed with FIJI/ImageJ (www.fiji.sc) using a custom macro. Briefly, images collected with Golgi in the first channel and Amylase in the second channel were collapsed as maximum intensity projections. Split channels were automatically contrasted with a saturation factor of 0.2 and converted to binary masks. Golgi and Amylase masks were auto-selected by white/black discrimination and area in µm^2^ calculated according to the meta-info embedded scaling factor. Percentage of Amylase outside of the Golgi was calculated by subtraction of the Golgi mask from the amylase mask divided by the total area of the Amylase mask. 40–45 cells were counted for each measurement. The number of Golgi stacks with amylase in a stack like morphology was counted manually for each cell. Zymogen granule area in hematoxylin and eosin stained paraffin sections was quantified by measuring the area occupied by zymogen granules and divided by the total cell area to give a % zymogen area. 7–10 acini were measured for each field captured on a 20x objective in a blinded fashion. Images were converted to. tiff format organized and processed using Adobe Photoshop CC (Adobe Systems, Mountain View, CA) for depicting in figures.

### Statistical Analysis

All values are presented as mean ± SEM. At least 3 independent experiments done for each data presented from *in vitro* studies. Differences between 2 groups were analyzed by unpaired Student’s t test while ANOVA (Dunnett’s method) was carried out to make comparison between continuous data from multiple groups. P value of <0.05 was considered to indicate statistical significance.

### Ethics statement

All animal experiments and relevant methods were approved by the Institutional Animal Care and Use Committee of the Mayo Clinic foundation.

## Electronic supplementary material

Supplementary Information
