# Supplementary material for: c-Src regulates cargo transit via the Golgi in pancreatic acinar cells
Source: Sci Rep. 2018 Aug 9;8:11903. doi: 10.1038/s41598-018-30370-4 (PMC6085363; doi:10.1038/s41598-018-30370-4)
Supplement: Supplementary file 1 — Supplementary Information [file 41598_2018_30370_MOESM1_ESM.pdf]

SUPPLEMENTARY INFORMATION

c-Src regulates cargo transit via the Golgi in pancreatic acinar cells

Sergiy Kostenko[1], Chan C. Heu[1], Jordan R. Yaron[1], Garima Singh[1], Cristiane de Oliveria[1], William J. Muller[2], and Vijay P. Singh[1]

[1]Department of Medicine, Mayo Clinic, Scottsdale, Arizona

[2]Goodman Cancer Research Center and Department of Biology, McGill University, Montreal, QC, Canada H3A 1A3

Corresponding author:

Vijay P. Singh, MD,

Associate Professor,

3rd floor Collaborative Research Building,

Mayo Clinic Arizona,

13400 Shea Boulevard, Scottsdale, AZ, 85259.

Phone: 480-301-4286

Fax: 480-301-7017

e-mail: singh.vijay@mayo.edu

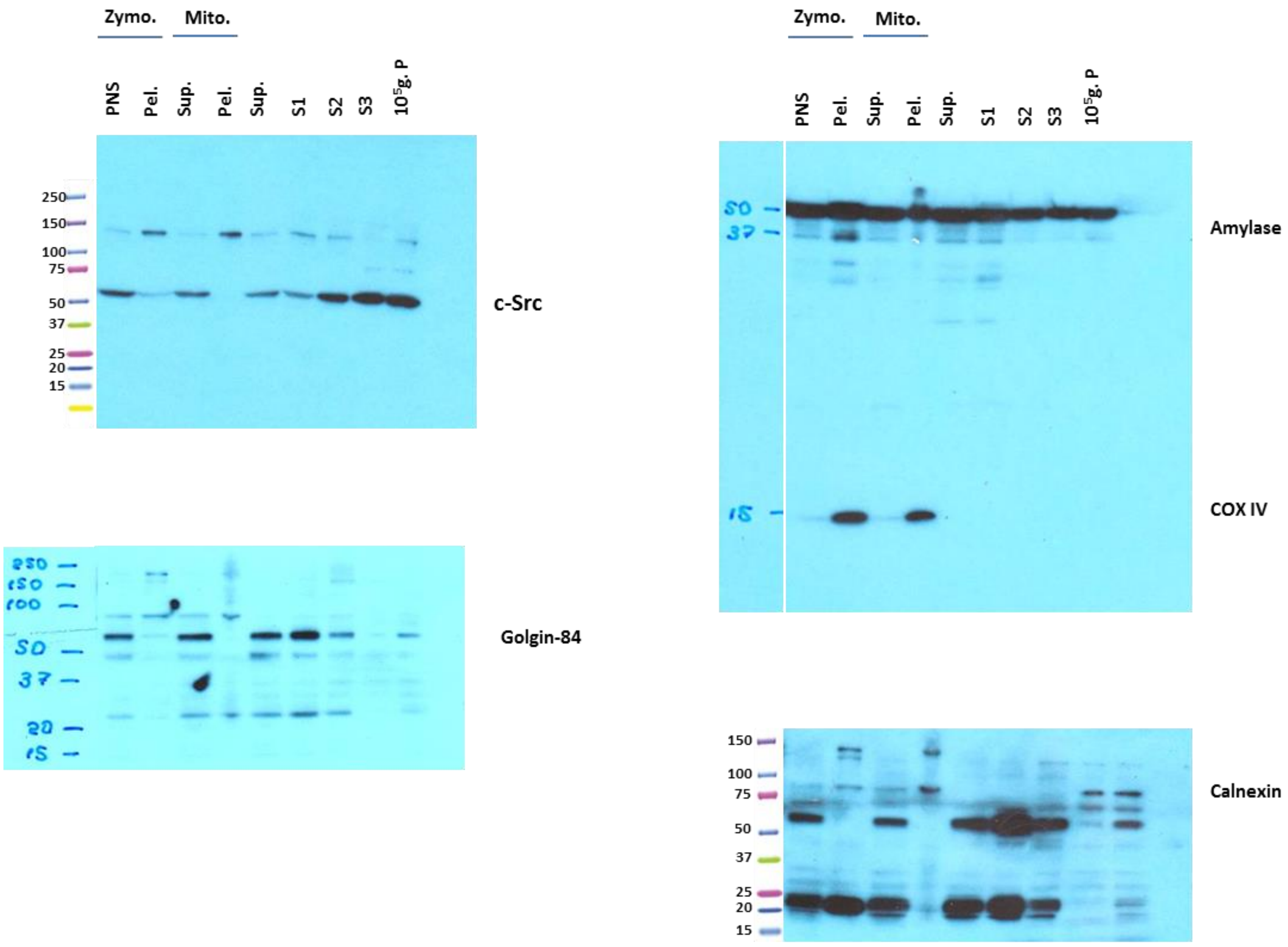

Supplementary Figure 1. Western blots of sub-cellular fractions of mouse pancreas separated into post nuclear supernatant (PNS), the Zymogen (Zymo.) pellet (Pel.) enriched in amylase and supernatant (Sup.), mitochondrial (Mito.) pellet enriched in COX-IV and supernatant, and microsomal fractions containing the Golgi marker Golgin-84 enriched in S1,S2 and the ER marker calnexin enriched in the S3, 10^5g. pellet fractions.

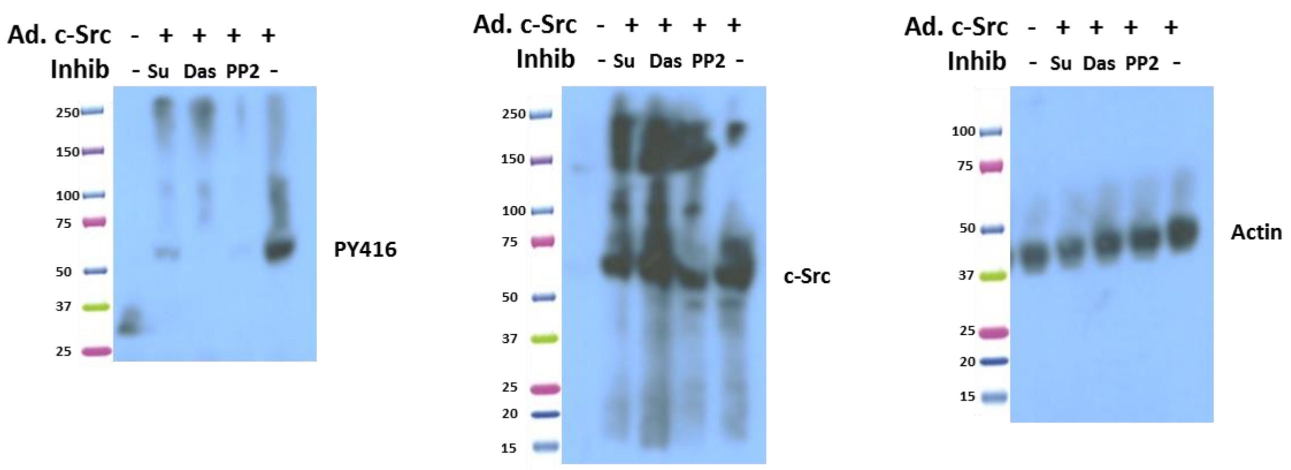

Supplementary Figure 2. Western blots of AR42J cell lysates overexpressing adenoviral c-Src blotted for active Src (PY416), c-Src and loading control actin showing the effects of pharmacologic inhibition of Src with SU6656 (Su), Dasatinib (Das) and PP2 on Src activation.

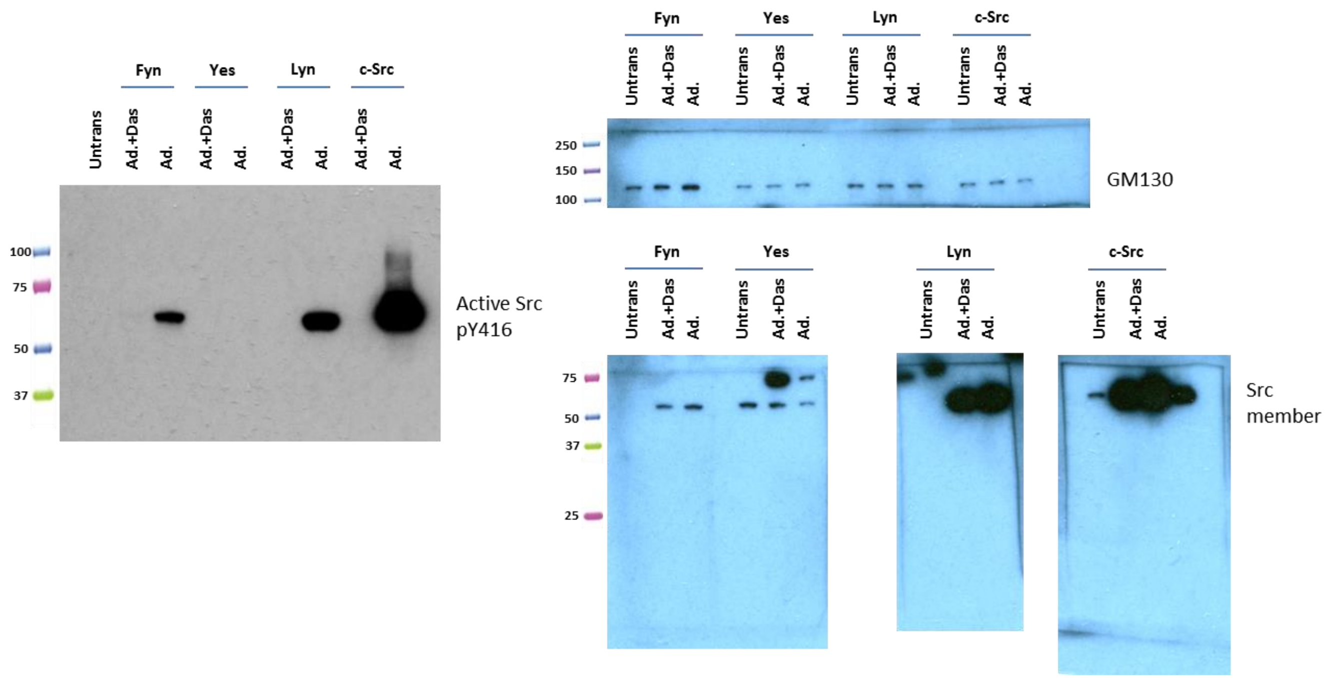

Supplementary Figure 3. Western blots of AR42J cell lysates overexpressing Fyn, Yes, Lyn and c-Src adenovirally with active Src (PY416), Src family member and GM-130.

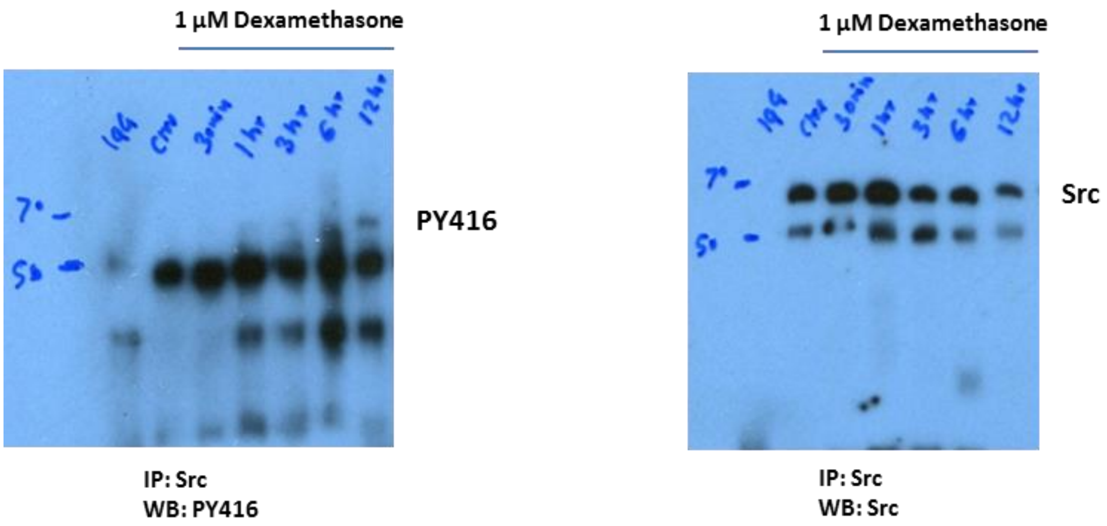

Supplementary Figure 4. Western blots of AR42J cell lysates collected after differ times of Dexamethasone stimulation immunoprecipitated for total Src and blotted for active Src (PY416) and total Src.

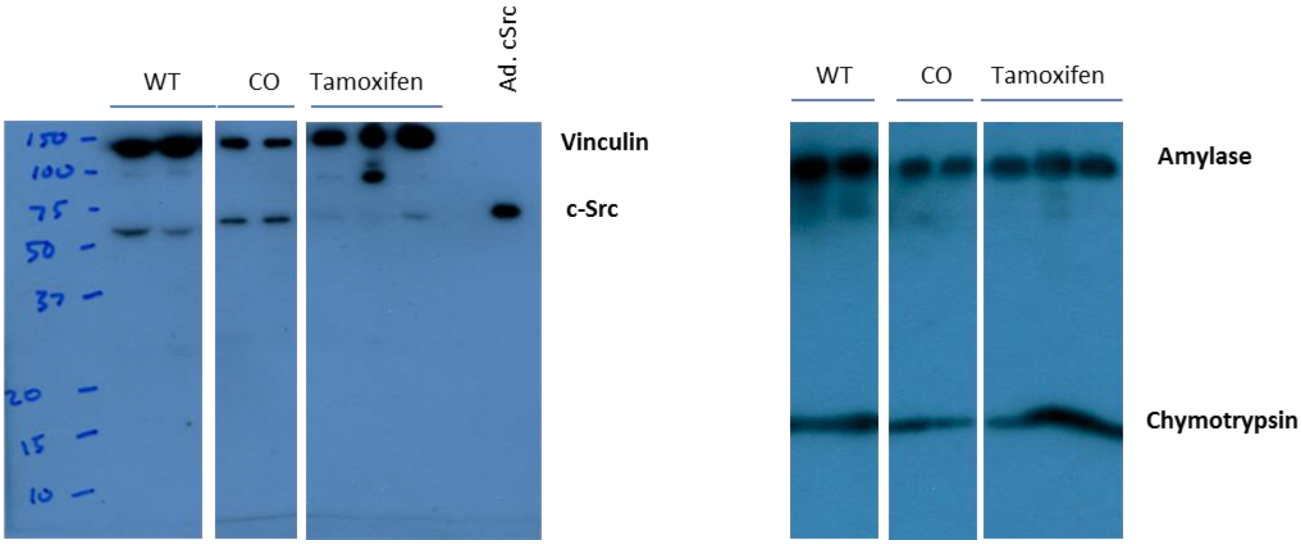

Supplementary Figure 5. Western blots of mouse pancreatic homogenates from C57BL/6 (wild type; WT) mice, Cre positive, c-Src L/L mice given corn oil (CO) or Tamoxifen blotted for c-Src (with c-Src adenovirus as a positive control), Amylase, Chymotrypsin and vinculin as a loading control.

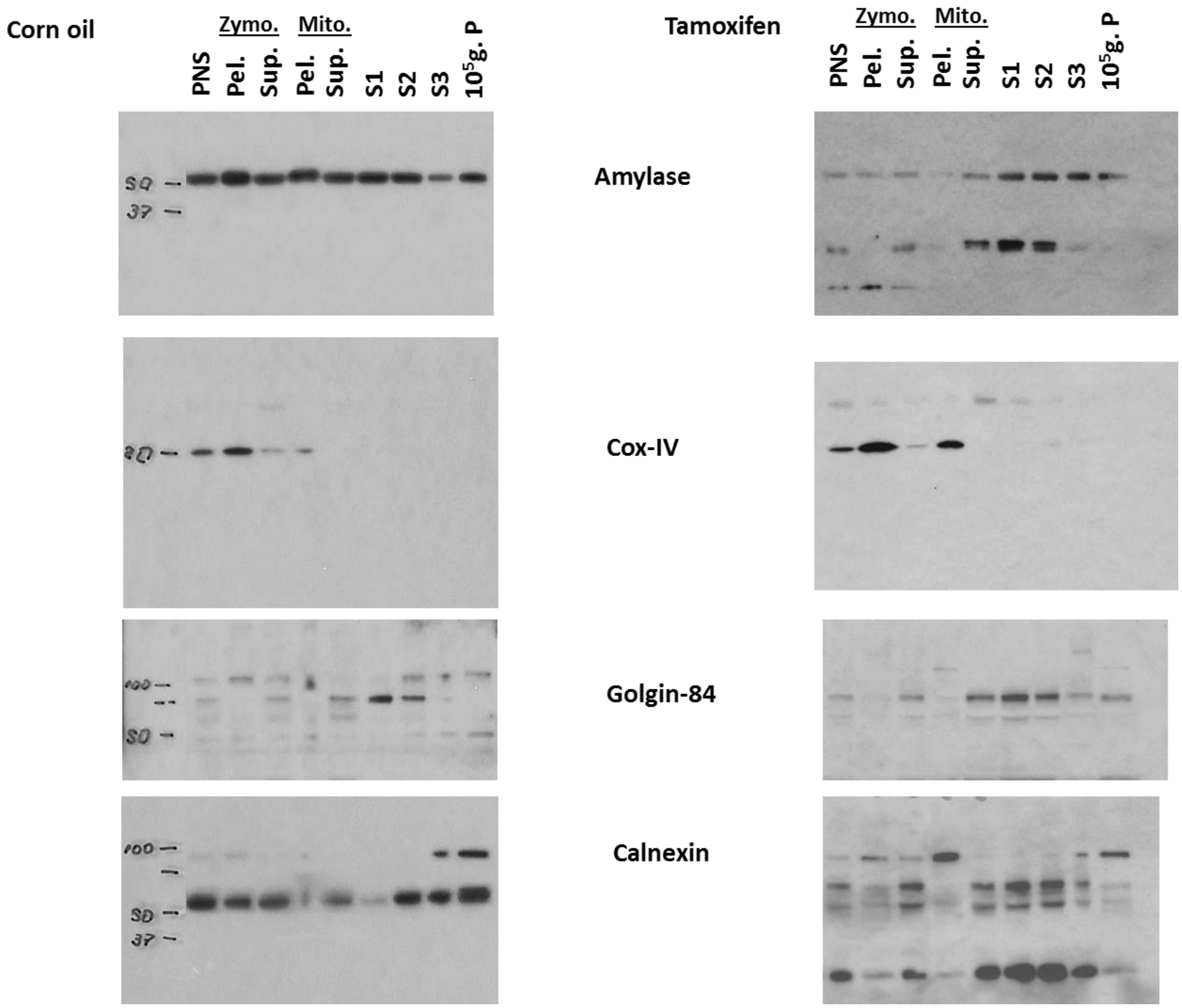

Supplementary Figure 6. Western blots of Subcellular fractionation of pancreas of corn oil and tamoxifen treated mice, blotted for amylase, along with the Golgi marker Golgin-84 and ER marker calnexin
